# Mining Dam Failures in Brazil: Comparing Legal Post-Disaster Decisions

**DOI:** 10.3390/ijerph182111346

**Published:** 2021-10-28

**Authors:** Paola Pinheiro Bernardi Primo, Michele Nacif Antunes, Ana Rosa Linde Arias, Adauto Emmerich Oliveira, Carlos Eduardo Siqueira

**Affiliations:** 1 Programa de Pós-Graduação em Saúde Coletiva, Universidade Federal do Espírito Santo, Vitória 29075-910, Brazil; micheleantunes@gmail.com (M.N.A.); adautoemmerich@terra.com.br (A.E.O.); 2 Escola de Matemática Aplicada, Fundação Getúlio Vargas, Rio de Janeiro 22250-900, Brazil; linde14@yahoo.com; 3 Public Health Master Programme, Graduate Entry Medical School, University of Limerick, V94 T9PX Limerick, Ireland; carlos.siqueira@umb.edu; 4 School for the Environment, UMass Boston, Boston, MA 02125, USA

**Keywords:** Mariana disaster, Brumadinho disaster, post-disaster management, legal decisions

## Abstract

Mining dam failures have increased worldwide since the 1980s. Two large mining dam failures occurred recently in Mariana and Brumadinho, both in the state of Minas Gerais, Brazil. We hypothesize that there were significant differences in legal post-disaster decisions. The aim of this article is to understand the similarities and differences of post-disaster actions and controversies in Mariana and Brumadinho. We reviewed 686 news reports about court decisions and settlement agreements from the websites of state and federal courts and judicial institutions. After classifying the reports using an adapted protocol from a media health observatory, we conducted a thematic analysis. Our analysis suggests that there were significant differences in legal post-disaster decisions in the cases of Mariana and Brumadinho. In Mariana, there was privatization of post-disaster management, with the creation of the Renova Foundation, a mediated indemnity program, lack of access to information for those affected, and uncertainties in health and resettlement issues. In Brumadinho, there was faster implementation of the recovery and compensation measures, faster recognition of affected parties, and stronger participation of the population since the first hearings. Even though there were particularities in post-disaster management, the ultimate goal of the corporations responsible for the disasters was to protect their profits.

## 1. Introduction

According to Pádua [1], the Brazilian intellectual tradition relegated environmental issues as secondary in political debates and limited them to superficial environmental discussions because that debate was assumedly foreign to Brazilian realities. Lisboa [2] was the first to produce an environmental critique in Brazil, based on Luso-Brazilian enlightenment philosophy. The first essay by Lisboa [2] included political reflections about the environmental development of the country and colonial gold mining exploration. Destructive environmental practices, rudimentary and unproductive agriculture, and mining exploration were dominant in that period. Pádua also commented on the work of Navarro [3] published in Portugal, wherein he perceived an amalgam of confrontational ideas and asked how the generosity of the land and men’s ingratitude were related and acted continuously to destroy, annihilate, consume, and weaken the substances of nature.

After observing the environmentally unfriendly practices of unskilled and poorly educated Brazilian miners, Lisboa [2] noted that to overcome that confrontation, new mineral production procedures and direction for mineralogy could be established in the country. He suggested that mining work be performed by environmental philosophers and magistrates to be more productive, as practiced in industrial nations in Europe where mines existed.

Navarro [3] criticized Portuguese colonizers who deforested trees for agricultural and basic subsistence production; after depleting the soils, they would settle in new lands. In addition, he supported lasting social and economic progress through smarter and environmentally friendly behaviors that would benefit all people and nature. On the other hand, Pádua [1] argued that the criticism was forgotten for centuries in Brazil. Since colonial times the mining practices adopted for mineral exploration and other economic activities, such as deforestation for agricultural production, were challenged. While the Brazilian colonial tradition critical of the country’s development is not capable of explaining all current issues, it allows us to contextualize the second decade of the 21st century, characterized by cruel and unjust socio-environmental and human realities. The mining disasters in Mariana in 2015 and Brumadinho in 2019, both located in the state of Minas Gerais (MG), are suitable examples of such realities

The dependence on the exploration and export of minerals in Latin American countries is a remnant of the colonial period. Brazil is one of the world’s largest iron ore producers and holds a large part of the world’s iron ore reserves. Most current large-scale iron mines produce significantly more wastes than in the past and also compared to other minerals. Mining tailings are stored in dams that are some of the largest engineering structures in the world and pose a high risk for fenceline communities [4,5,6].

According to the International Commission on Large Dams [7], all 221 mining dam failures reported between 1915 and 2001 could have been avoided because there was sufficient technical knowledge to keep the mining waste safely stored. In fact, mining dam failures have increased around the world since the 1980s. Vogel [8] notes that the annual rate of dam failures after 2000 has increased by five to six times. China is the country with the greatest number of these failures. Only Brazil and South Africa had more than one serious or very serious mining disaster in the same period [9]. In developed countries, there have also been mining dam failures, such as the failure of the Mount Polley mine dam in Canada in 2014, which is the biggest mining disaster in this country [10].

Similar factors may be responsible for the mining disasters in Mariana and Brumadinho, both in the state of Minas Gerais, where there were also failures in monitoring and emergency warning systems [9]. We hypothesize that there were also significant differences in legal post-disaster decisions that included socioeconomic, environmental, health, tourism, and cultural aspects, among others, in the cases of Mariana and Brumadinho. The aim of this article is to understand the similarities and differences of post-disaster actions and controversies in Mariana and Brumadinho.

In this article, we describe and compare the legal post-disaster decisions involving the corporations Samarco in Mariana and Vale S/A in Brumadinho. Our analysis was based on the four directives proposed by the Sendai Framework for Disaster Risk Reductions [11], focusing on recovery, rehabilitation, and reconstruction. We first present a comparative description of the two disasters, followed by the methodology chosen and the results of the comparison. Last, we discuss the results and offer conclusions on learned lessons from the most recurrent themes in settlement agreements and court decisions.

## 2. Materials and Methods

### 2.1. Mariana and Brumadinho: Similar Disaster Problems

**Figure 1:**
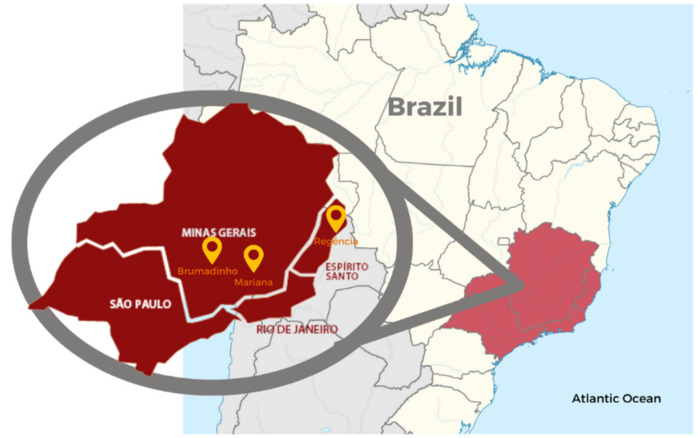
Map of Mariana and Brumadinho in Minas Gerais and Regência in Espírito Santo. (Source: adapted from the Brazilian Institute of Geography and Statistics (IBGE).

Mariana is a city located in the central region of Minas Gerais, where on 5 November 2015, the Fundão Dam, owned by the company Samarco Mineração S/A ruptured. Samarco is a joint venture between Vale S/A and B.H.P Billiton and one of the largest mining exploration conglomerates in the world. The rupture of the Fundão Dam is one of the largest environmental disasters in the mining sector in Brazil’s history. The dam failure released more than 40 million m^3^ of iron ore tailings, which destroyed important tributaries of the Doce River and contaminated 415 miles of the basin before reaching Regência in Espírito Santo and the Atlantic Ocean [12] (Figure 1).

The tailings flowed down the river, buried aquatic and terrestrial vegetation, killed animals, eliminated natural habitats by modifying and silting riverbeds, and greatly impacted cities in MG and ES [13]. According to the Bulletin of Civil Defense of MG [14], the rupture caused the death of 19 people and changed the lives of thousands of families who saw their homes, properties, health, and lives altered by the flood of toxic mud. Farah [15] noted that this disaster could have been even worse, according to a firefighter captain who was on site in the first fifteen hours post-disaster, because the emergency response teams were able to rescue over 700 residents despite the risk of rupture of a second and larger mining dam. After the disaster, people who lost their homes in Mariana were assembled in a gymnasium turned into an emergency shelter. Only after pressure from the State Prosecution Office (SPO) of MG were people relocated to hotels the following day. After a legal mandate, the company rented houses in Mariana in the ensuing weeks [16].

The dam disaster in Brumadinho occurred on 25 January 2019 when Dam B1, a tailings dam at the Córrego do Feijão iron ore mine, located nine kilometers (5.6 mi) east of Brumadinho, had a catastrophic failure. The dam is also owned by Vale S/A, one of the corporations involved in the disaster in Mariana. According to the national registry of the National Mining Agency (ANM in Portuguese), the Córrego do Feijão dam, built in 1976 by the Ferteco Mineração and acquired by Vale in 2001, was classified as a small structure with low risk of high potential damage. In December 2018, Vale obtained a permit to reuse the dam waste (about 11.7 million cubic meters) and to shut it down [17]. Dam B1 failed in January 2019, and the tailings flooded the Ferro-Carvão brook and reached the Paraopeba River. The force of the mud wave swept trains, vehicles, and ore processing machines in addition to the administrative center of Vale [18]. The tailings were mainly composed of iron, aluminum, manganese, and titanium but also had high levels of uranium, cadmium, lead, arsenic, and mercury [19]. They covered an area equivalent to 450 football fields along the river margins and may cause long-term negative impacts on plants, animals, and human beings [20].

In contrast to the Mariana case, there were 270 casualties in Brumadinho: 131 employees and 139 outsourced workers or residents of the nearby communities [21]. The high number of deaths occurred because Vale had built its office and a restaurant only 1 km (0.62 miles) downstream from the dam. The tailings hit those buildings a minute after the failure, which made evacuation impossible [22]. Despite Vale’s allegations that it had provided emergency response training to community members, residents did not have clear information about how to proceed in case of dam failure.

Institutional negotiations and agreements also had different dynamics in Mariana and Brumadinho. In the case of Mariana, in October 2016, the State Prosecution Office of MG publicized nine class-action lawsuits it had filed against Samarco in the Judicial District of Mariana. Thousands of individual lawsuits were also filed in both Minas Gerais and Espírito Santo [23]. Between November 2015 and December 2016, forty-four class-action lawsuits were filed against Samarco in both states. The federal government also became involved because the disaster impacted two units of the federation. As a result, a joint civil action lawsuit was also filed by the union and both state governments. In March 2016, the federal and state governments, various government agencies, Samarco, and its shareholders signed a consent decree (TAC in Portuguese), a legal document that stated the environmental obligations Samarco must comply with.

This extrajudicial agreement created the Renova Foundation, a private foundation governed by an executive office, which would answer to a board of trustees, an advisory board, and a fiscal council. External monitoring and control fell under the responsibility of auditors chosen by the foundation, the advisory panel of experts, and the inter-state committee (IFC in Portuguese), a body comprising representatives of government departments [23]. The TAC defined Renova Foundation as the main organization responsible for implementing “measures for recovery, mitigation, remediation and reparation, including compensation” [24]. The foundation’s bylaws established that the board of trustees was responsible for selecting the executive office, approving internal regulations, and determining the correction of any irregularities in the operation of the foundation, among other duties. The board is made up of seven members, of whom only one is an independent member appointed by a special committee. Representatives of the population affected were only included in the governance structures of Renova in June 2018, over 2 years after its creation [25].

In the case of Brumadinho, there was a meeting on the same day of the disaster involving different legal institutions to discuss collaborative measures [26]. Impacts on the São Francisco River, which flows from MG to the state of Bahia, were not identified then; therefore, there was no justification for the involvement of federal institutions in legal procedures. The State Public Ministry (SPM) and the Federal Public Ministry (FPM) joined as Amici Curiae [27] in a lawsuit that allowed them to participate in all hearings and stakeholder meetings. According to the Ministério Público Federal (MPF) website, “The MPF acts on those federal matters regulated by the Constitution and federal laws whenever public interest is involved, by virtue of the parties or of the matter itself. It is also the MPF’s responsibility to ensure compliance with the laws in force in Brazil, including international agreements. Furthermore, the MPF acts as a guardian of democracy, ensuring respect for principles and rules that guarantee popular participation.” [27]. Another translation to MPF is Federal Prosecution Service. Similar to the MPF, the Ministério Público (MP) acts on public interests at the state level. 

Legal institutions rejected the involvement of the Renova Foundation in post-disaster management. Instead of an extrajudicial agreement, Vale and the legal institutions opted for mediated negotiations. There were many meetings between the parties, and agreements were discussed in public hearings before a judge. Unlike the Doce River case, where Renova Foundation produced all data, the judge hired the Federal University of Minas Gerais (UFMG) at Vale’s expense to collect evidence about social and environmental damages [27].

There was no direct community participation during legal procedures because legal institutions (particularly the SPM-MG) officially represented affected communities. By the end of February 2019, those legal institutions requested that impacted people or their representatives be present in the hearings. When attendance increased in March, the judge complained that a high turnout created “difficulties” to procedures and required individuals to register in advance. When community representatives were present, they had to sit behind a glass window and could not openly display their demands (e.g., banners and placards were forbidden). Later, attendance criteria became stricter. The judge required the Brazilian Bar Association to nominate five lawyers to be allowed to attend hearings in the courtroom. As people who were not included demanded participation, the judge decided that community representatives had to take turns in attending hearings [28]. As a result, people’s attendance and participation were restricted, which created great dissatisfaction in local communities.

### 2.2. Data Sources

Our data sources included official news reports about court decisions and settlement agreements published on the websites of the following state and federal courts and legal institutions: State Court of Minas Gerais (TJMG) and Espírito Santo (TJES), Federal Public Ministry (MPF), and State Public Ministries of Minas Gerais (MPMG) and Espírito Santo (MPES). Those legal institutions were chosen because they were in charge of deciding on post-disaster legal remedies to punish environmental and workplace violations, compensation for affected parties, and payment for environmental recovery associated with the dam ruptures in Mariana and Brumadinho.

**Figure 2:**
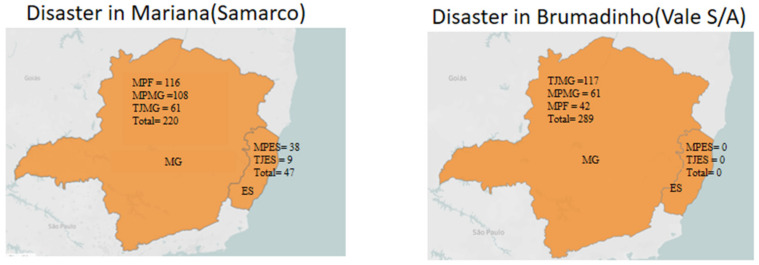
Number of reports of the disasters in Mariana and Brumadinho (source: authors).

We used the SigDesastre Information System to collect information from the official MPES and TJES websites [29]. The SigDesastre is an Internet information monitoring system. The objective of SigDesastre is to monitor information about Mariana’s disaster. The use of information monitoring on the Internet is considered an important tool for the post-disaster risk communication process [29]. To collect information from the TJMG, the MPF, and the MPMG, we conducted searches in their respective websites using the search terms “Brumadinho OR, Vale S/A, Mariana OR, Samarco.” We reviewed 686 news reports. After a cursory reading of the reports, we excluded those that did not address lawsuits or follow up of settlement agreements and mediation hearings. Figure 2 shows the distribution of news reports selected for further analysis.

**Figure 3:**
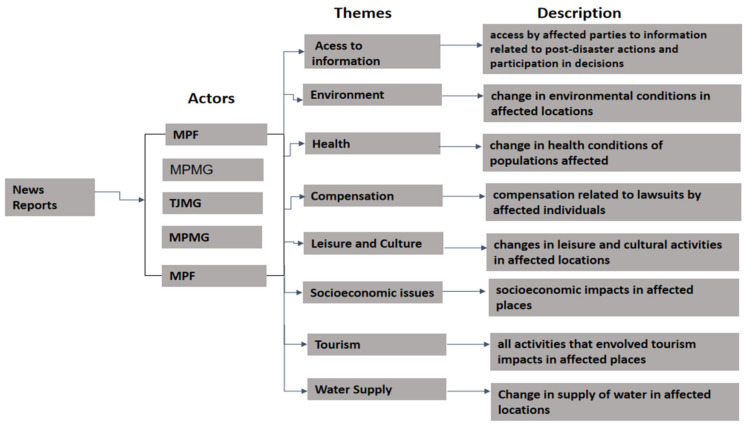
Schema of analysis protocol (source: authors).

Next, the selected reports were read in detail, and sections of the texts related to chosen themes were recorded in Excel spreadsheets and texts grouped according to themes (Figure 3). We followed protocol adapted from the Regional Media Health Observatory of Espírito Santo (OSM-ES), a project developed by the Collective Health Program (PPGSC) of the Federal University of Espírito Santo (UFES) and the Institute for Communication of Scientific and Technological Information of the Institute Oswaldo Cruz Foundation in the city of Rio de Janeiro (ICICT/FIOCRUZ). The protocol had been used in other disaster studies [30] as well as studies on diabetes mellitus [31,32,33] and neglected diseases.

The protocol used the following categories to classify the reports: source of report, data of publication, report title, site page, social actors involved, themes addressed, and report section (Table 1).

## 3. Results

### 3.1. Quantitative Thematic Analysis

**Figure 4:**
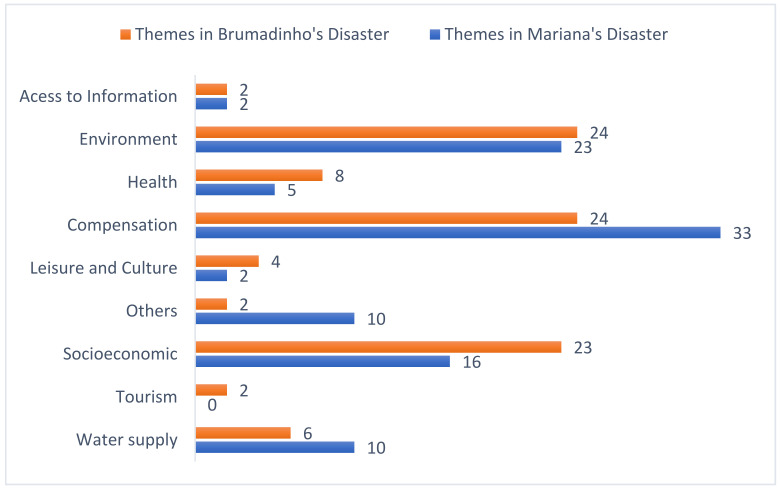
Relative distribution of themes in news reports.

The quantitative description of themes according to their frequency in reports about settlement agreements and court decisions is shown in Figure 4. The most common theme in news reports about both disasters was compensation, corresponding to 24% of news reports in Brumadinho and 33% in Mariana, followed by environmental issues. Health, tourism, leisure, and culture are mentioned less in both disasters. In Brumadinho, they are mentioned in 8%, 2%, and 4% of the reports, respectively. In Mariana, health is mentioned in 5% while leisure and culture are mentioned in 2% of the reports, and tourism was not at all mentioned. The themes of compensation and environment are the most frequent in both disasters.

**Figure 5:**
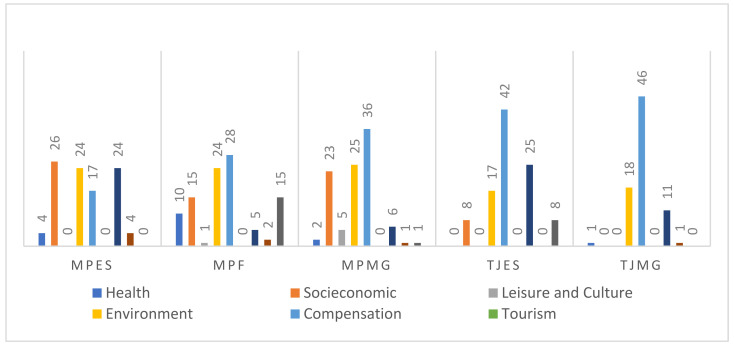
Relative distribution of themes in the disaster in Mariana according to legal institution.

When themes were analyzed according to the institution that issued the legal decisions about the Mariana disaster, we noted a marked difference in frequency between the two disasters. The MPES addressed lawsuits about water supply (24%), but the news reports also mentioned water quality in the Doce River basin. The TJMG, on the other hand, addressed compensation issues more often (46%), as indicated in Figure 5.

**Figure 6:**
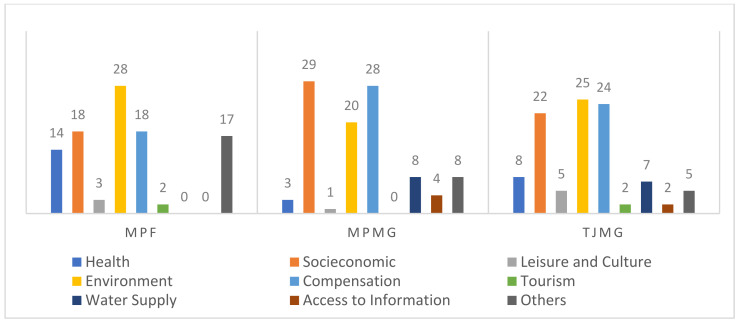
Relative distribution of themes in the disaster in Brumadinho according to legal institution.

When we conducted the same analysis for the disaster in Brumadinho, compensation was frequent, though it was not the most common theme. The MPF, for example, emphasized environmental issues (28%), while the MPMG focused on socioeconomic issues (29%), as shown in Figure 6.

### 3.2. Timeline of Disaster Themes

**Figure 7:**
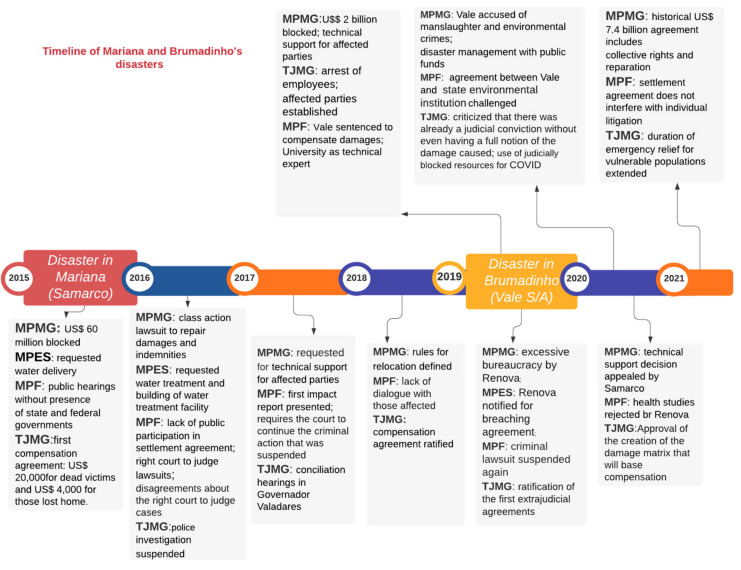
Timeline of disasters (sources: authors).

After conducting descriptive statistical analysis, we developed a timeline for post-disaster lawsuits and settlement agreements to assess when they appeared in the reports, which might indicate changes in post-disaster debates, and the chronology of implementation of legal decisions in the two cases (Figure 7). This timeline is based on the main events that occurred between 2015 and 2021 in Mariana and between 2019 and 2021 in Brumadinho.

This timeline reveals that the dam failure in Mariana led to legal decisions to block assets of Samarco, concerns about the environmental impact on the Doce River basin and water supply to the counties affected, and the beginning of settlement agreements with the population of those counties. After 2016, other themes concerned legal institutions, such as jurisdictional disputes, institutional agreements on compensation values, and the creation of the first consent decree involving federal and state governments, corporations, and environmental agencies. Since representatives of local communities were not included in the discussions about the terms of the decrees, which established the creation of the Renova Foundation to manage victim compensation resources, the courts did not endorse the decree.

After 2017, technical assistance was often mentioned in the news reports reviewed. Consultants helped the courts and the local communities to decide on post-disaster measures. In 2018, the focus of the reports changed to the inclusion of affected communities in hearings and in the administrative structure of the foundation, as well as establishing the TAC-Governance. This TAC created the Mediated Compensation Program (PIM in Portuguese) to compensate communities and micro and small enterprises that “suffered losses or damages related to their economic activities as a direct result of the Fundão Dam failure in Mariana, without bureaucracy and the cost of a lawsuit” [34]. This program compensated local residents who lost income or assets and residents in the counties whose supply and distribution of water were temporarily interrupted by the disaster in Mariana [34].

In 2019, news reports focused on discussions about compensation values, conciliation hearings about water supply, especially in the city of Governador Valadares (MG), and rebuilding the village of Bento Rodrigues, renamed as New Bento. In 2020, after five years of the Mariana disaster, there were still debates about health studies, hiring of technical advisors and the results of studies conducted, and even the creation of the Renova Foundation.

In Brumadinho, post-disaster managerial procedures were different from Mariana. The legal institutions involved took different positions in several issues, such as the determination of affected parties, the inclusion of community representatives from the beginning of post-disaster litigation discussions (though this did not mean that they effectively participated in it), and support for health, economic, and environmental needs. Conciliation hearings were held throughout 2019 to negotiate settlement agreements with indigenous communities, families who lost loved ones, and affected communities and counties, in addition to the participation of faculty and staff from the UFMG as expert consultants.

As far as corporate responsibility is concerned, employees of Vale S/A and the German engineering company TüdSüd, responsible for auditing the dam in Brumadinho, were held accountable and charged with criminal misconduct. Employees were detained for a few months for further investigation. The same accountability did not happen in Mariana. At the beginning of 2021, after monthly conciliation hearings, a historic settlement agreement in the amount of USD $7.1 billion was signed between Vale S/A, state, and federal institutions. The agreement “aimed at socio-environmental and socioeconomic reparations as an anticipation for compensation of damages” [35].

In the case of Mariana, the criminal prosecution was on hold for over two years, despite the complaint filed by the Office of the Public Prosecutor. Only six of those charged remain as defendants [36]. In the case of Brumadinho, the MPMG argued that Dam B1 of the Córrego do Feijão was unsafe from the geotechnical perspective and a situation well known by Vale management. The lawsuits produced reports indicating that some of the technical employees and consultants were aware of the risks posed by the dam but were negligent in adopting available and known measures addressing transparency, security, and emergency. Therefore, they were responsible for the deaths and environmental damage resulting from the dam failure [36]. In February 2020, the Criminal Judge of the Second Criminal Court of Brumadinho received a complaint from the Office of the Public Prosecutor regarding the failure of Dam I. Sixteen people were charged, among them the then CEO of Vale, Fabio Schvartsman, as well as other directors, managers, geologists, and consultants of the corporations Vale S.A. and TüvSüd Bureau of Projects and Consultancies.

### 3.3. Comparative Analysis

Table 2 compares the post-disaster themes derived from the news reports of legal decisions according to their frequency in those reports. It shows that hiring technical consultants was mentioned in both the health and access to information themes. Based on the experience with the Renova Foundation in Mariana, legal institutions supported the need to provide technical assistance for impacted communities in Brumadinho, which should include delivery of information understandable to those communities, as well access to planning, execution, evaluation of the matrix of damages and reparation [37]. Nevertheless, Vale repeated the delaying tactics of the foundation and also resisted technical assistance by imposing conditions and rejecting established work plans. Thus, the technical assistance started later.

## 4. Discussion

The mining disasters described in this article have similarities not only because both are mining dam failures but also due to similar outcomes. Both led to a great loss of life, environmental pollution as well as triggered citizen lawsuits and penalties against Vale S/A for violation of administrative procedures enforced by the Brazilian organization responsible for regulating securities and exchanges (CVM in Portuguese). Both disasters also caused federal and state prosecutors to file civil action lawsuits and establish consent decrees that included state governments, corporations, and state departments of the environment [38]. Both disasters were also workplace accidents because they originated in mining production processes, interrupted work, and interfered in daily activities and lives of workers and communities. In sum, they caused great human, economic, environmental, and health impacts in local populations, overwhelming the response capabilities of the communities, counties, and regions directly affected. Some Brazilian authors call such types of disasters expanded workplace accidents [22].

Rojas and Pereira [39] discuss the institutional and legal architecture deployed by corporations to manage the disaster in Mariana and argue that their usual operational procedures oscillated between two poles. On the one hand, their emergency response, compensation, and reparation actions in the affected areas were slow, absent, and negligent. On the other, they acted fast to reshape institutional and legal apparatuses to back their corporate interests. Valencio [40] adds that when a disaster occurs, corporate legal departments intervene immediately to make sure that corporate interests are preserved and to prevent losses that may harm the financial viability of the corporations. Those departments are the legal structures that protect corporate mining interests. This is clear in the disaster timeline, where one can infer the slow pace of compensating affected parties and determining eligibility for compensation.

Although the quantitative analysis suggested that the frequency of themes in both disasters were similar, the qualitative analysis suggested otherwise. Differences in themes mentioned in both disasters became notable.

### 4.1. Compensation

In Mariana, the consent decree that established the creation of the Renova Foundation, a private concern, and the mediated compensation programs demonstrate the innovative and sophisticated legal and institutional architecture organized by the corporations to manage post-disaster damages. Thus, at the macro level, a number of agreements were negotiated through the TACs, whose stipulations have been continuously ignored by the corporations. At the micro level, compensation of affected parties was negotiated at the individual level and made difficult by victim registration methods incompatible with the context of local residents. Three years after the disaster, news reports mentioned that many people still waited to be recognized by the courts as affected parties [41], and those who were had to face the mediated negotiation adopted in the PIM.

The strategy used by the Renova Foundation to negotiate the compensation of individuals through mediation increased the existing inequality between the parties because the ability of poor residents to negotiate fair compensation for losses and damages was quite limited due to their urgent survival needs [42]. In addition, the PIM adopted procedures that made full reparation difficult since it did not prioritize individual needs and required a great deal of documentation from claimants [43].

Samarco’s management of the registry of claimants and the Emergency Relief Program were denounced early by public prosecutors for their lack of transparency regarding eligibility criteria for emergency relief and differential treatment of residents. The lack of transparent criteria for emergency relief created conflicts in the communities and left many people clearly impacted by the disaster without any compensation [27]. Although compensation was a common theme in news reports, legal decisions did not target those directly affected by the disasters, especially vulnerable residents.

The PIM was also criticized by social movements and civil society organizations, who claimed that corporate interests had captured the judicial branch and the set of tools and mechanisms available within the Brazilian legal system. According to authors such as Rojas and Pereira [39], all procedures adopted by Samarco, Vale, BHP, and the Renova Foundation were favorable to their interests, including the extension of deadlines to compensate residents, settlement agreements with courts to speed up and standardize the amount of compensation to individual claimants, postponement and revocation of deposits worth millions of dollars, maintenance of abusive clauses in the agreements, suspension of over 50,000 lawsuits filed against the corporations, among others. According to the report of Cáritas Brasileira Regional MG [44], no family had been relocated by early 2021, which is a violation of people’s dignity and life conditions as they became permanently homeless.

In the case of the disaster in Brumadinho, our timeline shows faster outcomes deriving from the participation of legal institutions in the governance model adopted to discuss collaborative institutional agreements [45]. The legal institutions involved quickly rejected any participation of Renova Foundation in decisions, aiming at not repeating the experience of the disaster in Mariana. In the first meetings with the corporations responsible for the disasters, legal institutions and Vale opted for a negotiation that would end up in a settlement agreement instead of prolonged litigation. There were conciliation meetings between the parties that included the state of Minas Gerais, legal institutions, and representatives of the communities affected. All progress was presented in public hearings held by a judge. In contrast to the Mariana case, where Renova Foundation hired private consultant companies to produce data and matrices to quantify damages, in Brumadinho, a judge determined that all evidence on social and environmental damages be collected by academics and staff led by the UFMG [46].

The compensation agreement of February 2019 in Brumadinho, less than a month after the disaster, established an emergency relief payment for impacted communities as a way of preserving their income. All residents in Brumadinho and those who lived up to 1 km from the river margins received a monthly payment during the following 12 months. This agreement differed from the one in Mariana since in Mariana, the Renova Foundation was in charge of deciding who was eligible for financial support through special forms and numerous criteria, while in Brumadinho, geographic criteria prevailed. Furthermore, in the latter, the payment was per capita, while for families who lived alongside the Doce River, payment was per family unit [37].

### 4.2. Access to Information

There was limited participation of impacted communities in decision-making in both disasters.

In the case of Mariana, there was some representation in the governance of the Renova Foundation, but only after changes in the TAC-GOVERNANCE in 2018 did two community representatives become part of the Board of Curators, and four were accepted in the advisory board of the foundation [47]. However, according to Losekann and Milanez [48], this limited participation may end up contributing to legitimizing decisions made previously in a bureaucratic and tangled structure. This gap in democratic accountability may explain why five years after the dam failure in Mariana, the impacted communities of Bento Rodrigues, Paracatu de Baixo, and Gesteira received little resources. Despite their representation in those boards, they still were a minority among representatives of the corporations that caused the disaster.

Furthermore, the hierarchic structure of several technical subcommittees, councils, the inter-state committee, beyond the many challenges of the foundation about results of studies conducted by experts, produced endless debates and delays, a situation that could be summarized as “paralysis by analysis” [49]. In turn, those delays had repercussions on the proposed schedule for post-disaster management

In the case of Brumadinho, communities were invited to participate in the first meetings and hearings but were surprised by the limited number of people allowed to attend them. As a result, many were not able to have access to decisions. Those restrictions increased the influence of legal institutions and reduced the power of communities to shape the results of negotiations. Even though community representatives were strongly in favor of collective negotiations because they understood that they would be disadvantaged if families had to negotiate separately with attorneys from Vale, in February 2019, Vale demanded that negotiations had to be conducted with each impacted family [26,50].

### 4.3. Health

While health issues were urgent, the theme was not common in legal actions in both disasters (Figure 4). Yet, studies by consultants indicated that there was general insecurity regarding water supply and fishing as well as the risk of consuming fish. Those issues were the focus of continuing questions by the population, which remain unanswered since there still are no conclusive studies on the health risks faced by the population that resides in the Doce River basin.

The affected populations did not trust the information and technical reports published by the foundation, which kept communicating “the nonexistence of causal associations between the disaster and the majority of symptoms and disorders identified in the population” [42]. In the Brumadinho case, data from the Ministry of Health indicate an 80% increase in the consumption of anxiolytics and an increase of 1.272% in reactions to serious stress in 2019 compared to 2018, which suggest that those disasters affected both the physical and mental health of communities impacted [22].

The disasters reviewed in the news reports caused incalculable public health impacts. According to Freitas et al. [9], lessons learned in the disaster in Mariana about environmental impacts, risks, damages, diseases, and corporate and health sector response contributed to the delivery of a set of emergency and integrated services by the Brazilian health system (SUS in Portuguese) in the disaster in Brumadinho. The authors claim that those services have decreased risks of disease and expanded surveillance and health care activities [9].

### 4.4. Socioeconomic

Our analysis suggests that in the Mariana case, Samarco bet on managing the reparation of damages through corporate management lenses [39] as a strategy to control the environmental reparation costs of the Doce River. It privatized procedures via mediated compensation and the creation of the Renova Foundation. Since those were weak solutions, they removed the corporation as a responsible party for compensating socioeconomic and socio-environmental damage to the communities in the basin. The corporate management of the post-disaster, led by the foundation, had as its main goal to let Samarco off the hook. The foundation became the main actor, if not the single, in managing post-disaster risks, which made the corporation itself the driver in compensating the damages that it caused and reduced the role of the state [48]. The strategies used by Samarco to address the post-disaster crisis were not successful. Samarco was only authorized to restart operations by the end of 2020. In April 2021, it filed for judicial recovery with debt over USD $10 billion [51]. On the other hand, Vale appears to be successful. Two years after the disaster, a settlement agreement was approved, and despite the celebration by legal circles and the state, the value agreed upon for compensation was half the amount initially requested, around USD $12 billion. A year later, Vale had already recovered its market value. In the first trimester of 2021, it had a net profit of USD $6 billion [52].

In the Brumadinho case, the evidence presented in news reports confirms that Vale knew that the Fundão Dam had structural failures that could have been mitigated through adequate drainage [53]. The construction of the employee cafeteria below the dam and lack of response to risks identified by external auditors are examples of gross negligence and willful misconduct in minimizing risks [26].

### 4.5. Lessons Learned

Based on the directives proposed by the Sendai Framework [11], the first lesson from the disasters caused by Samarco and Vale is that Brazil has been incapable of making progress in policies and procedures to reduce the risk of mining dam failures. This same document also recognized the role of private institutions in reducing risk in disasters, including tailings dams and their potential risk to surrounding populations [52]. Freitas et al. [9] call the management of risks in mining disasters in Brazil “artificial management of risks”, which turn abnormal into normal risks. In this management policy and style, government departments and corporations try to build an image that they effectively prevent and control the risk of accidents while at the same time try to quell discordant voices of workers, social movements, unions, and associations that insist on speaking up against injustices and misconducts.

The news reports also suggest that mining corporations were the dominant power while the state allowed and participated in solutions that did not address the inequalities between the parties. In addition, the Brazilian environmental legislation has been weakened, and there was a lack of investment and shortage of human resources in the National Mining Agency, which is responsible for inspecting mining dams in Brazil [54]. Although corporate managers were aware of all risks, they opted for continuing operations. This disregard for potential risks combined with lack of popular participation in decision-making in both disasters create what Tierney [55] calls corrosive communities, that is, communities that suffer after disasters “by lack of consensus and by controversies: there is no collective definition of the situation; there may be legal litigation processes; there is no closure of the situation; and the attribution of blame and the appointment of those responsible emerge, with the inherent lack of trust in institutions and people” [56]. Such experiences create in individuals what Freudenburg and Jones [57] call “recreancy”, or a lack of trust in institutions and the state apparatus.

Those management policies and practices are standard operating procedures for disaster risk management in the mining industry in Brazil. The less risk is known, the greater the corporate power over fenceline populations to build and operate hazardous facilities such as tailings dams. On the other hand, transparent risk communication may help local populations in decisions about how to live in a territory with a potential risk of disaster. Disclosure of risks and transparency can also help governments approve mining projects.

Our results indicate that the disasters in Mariana and Brumadinho uncovered poor risk management and oversight of iron mining practices as well as post-disaster mismanagement. While there were significant differences in post-disaster measures, the main source of socio-environmental problems is the same, i.e., lax mining exploration policies adopted in Brazil since colonial times. Those lax policies were poorly enforced by the state over the years and became toothless during the last two decades when neoliberal Brazilian governments strengthened the country’s dependency on exports of raw materials [58,59,60].

Our study has strengths and limitations. We accessed official news reports from the legal institutions in MG and ES, which are based on a large amount of evidence provided to the courts. Legal standards of proof had to be followed by all parties involved. The evidence had to be substantiated by scientific findings and testimony of a broad variety of organizations and individuals, which strengthens the reliability and validity of the data and information available in the news reports reviewed. However, we were not able to interview stakeholders involved to obtain an in-depth perspective about the interests at play in the meetings and hearings held in both disasters.

## 5. Conclusions

We analyzed in this article the differences and similarities in post-disaster management according to news reports of legal actions taken in the disasters in Mariana and Brumadinho. Our study suggests that there were significant differences in legal post-disaster decisions in the cases of Mariana and Brumadinho. In Mariana, there was privatization of post-disaster management, with the creation of the Renova Foundation, a mediated indemnity program, lack of access to information for those affected, and uncertainties in health and resettlement issues. In Brumadinho, there was faster implementation of the recovery and compensation measures, faster recognition of affected parties, and stronger participation of the population since the first hearings. Yet, speeding up individual settlement agreements, reducing the lag time to compensate losses and damages, and including community representatives in discussions and decisions did not substantially change the usual mining production procedures and storage of mining wastes nor provided adequate resources to the affected populations.

In both cases, we observed the same mining production procedures based on dilapidation of mineral and environmental resources, exploitation of labor and communities, and manipulation of the state and its enforcement procedures. Samarco and Vale are powerful polluting corporations able to obtain flexible public policies favorable to their commercial interests; nature is thus treated as a commodity and source of profits above all [58,60].

Our study contributes to increasing the understanding of post-disaster legal decisions and agreements and a better understanding of dam tailings post-disaster management and the consequences for nearby communities. We hope that future studies deepen the understanding of post-disaster legal decisions, including the perspectives of the multiple parties involved in post-disaster litigation.

Our conclusions are similar to the one stated by Feitosa, a resident in the local district Juatuba:

“The agreement only rewards the mining corporation Vale, which solves a problem with shareholders, leaving affected people and their demands out of it. This once more demonstrates that Vale is rewarded. It is already a repeat offender for the crime that occurred in Mariana. It killed additional 272 people, and again it comes out on top; it comes out well, doing whatever it wants, and we feel that the State is not concerned with doing justice”, testimony of Ms. Joelisia Feitosa, resident in Juatuba [61].

## Figures and Tables

**Table 1:** News reports themes and passages.

Theme	Example *
Access to information	“The MPES also guides public organizations, fisherfolk and environmental associations to, within their respective jurisdictions, collect and store information, photos, footage, documents and other elements that support eventual losses” (MPES, 7/11/15)
Environment	“...and the Federal Public Ministry (FPM), filed in Federal Court a Civil Action Lawsuit (ACP)...aiming at collecting and analyzing seawater near the Doce River...” (MPES, 11/11/2015)
Health	“Foundation Oswaldo Cruz (Fiocruz) will implement epidemiological surveillance actions and Foundation Ezequiel Dias (Funed) will follow up and monitor blood samples of all living beings to detect levels of heavy metals ” (MPMG, 4/4/19)
Compensation	“Objective is to find out the reasons for excessive delay in the analysis and approval of financial relief requests by people who, while not providing documentation of occupation as fishermen, earned their income by fishing” (MPF, 1/7/2020)
Leisure and Culture	“the losses are huge and irreversible. He refers mainly to a chapel in Bento Rodrigues, built in the XVIII century, which goes back to the origins of the village and is totally buried under mud.” (MPMG, 11/06/15)
Socioeconomic issues	“Foundation Renova has refused to compensate real estate damages caused by removing mud that covered sections of Barra Longa City (MG) after the rupture of the Fundão dam” (MPF, 6/4/20)
Tourism	“... were listed by the Public Defense Office, which chose the names among farmers, merchants, maroons, microentrepreneurs and fisherfolk” (TJMG, 7/8/19)
Water Supply	“The Judicial Center for Citizenship and Conflict Resolution (Cejusc) of Governador Valadares homologated in 2017 a total of 25.550 agreements between Renova Foundations and county residents regarding complaints for water supply in the region” (TJMG, 1/23/18)

* free translation by authors.

**Table 2:** Comparison of post-disaster themes in Mariana and Brumadinho.

Theme	Disaster in Mariana	Disaster in Brumadinho
Compensation	➢ Emergency relief payment to the people affected after registration.	➢ Emergency relief payment to all residents of Brumadinho and those who lived within 1 km of the margins of the Paraopeba River.
	➢ Mediated Compensation Program created by Renova Foundation. Programa discussed because it included unfair clauses.	➢ Agreement for collective and diffuse damages in the amount of USD $ 7.1 billion. Awards of USD $2.3 million for those who lost relatives.
Environment	➢ Disagreements over permission for fish consumption.	➢ More pressure on government environmental and inspection departments responsible for mining dams.
	➢ Until 2021, no environmental project finished.	➢ Hiring of independent consulting to evaluate other dams owned by Vale.
	➢ Suspension of environmental permits of Samarco for four more years.	➢ After 2 months, courts authorize Vale to reopen.
	➢ New bill to increase inspection of dams.	
Socioeconomic	➢ USD $60 million blocked for compensation of damages.	➢ USD $2 billion blocked for compensation of damages.
	➢ Consent decrees issued included 42 projects.	➢ Technical advice for the population affected.
	➢ Renova Foundation created to manage post-disaster measures.	➢ Preliminary consent decree to accelerate recovery projects.
	➢ No program finished by 2021.	➢ Agreement in the billions two years after disaster.
Water Supply	➢ Poor water quality for drinking and fishing.	➢ Vale agrees to supply water for counties affected. Pays for technical consultants to monitor water quality.
	➢ Water supply for affected counties.	➢ UFMG hired to provide technical advice.
	➢ Many lawsuits alleging moral damage for lack of water supply.	➢ Construction of alternative water collection from the Paraopeba River.
	➢ Still lack of consensus about the water quality of the Doce River.	
Health	➢ Hiring of technical experts to conduct health risk assessment only occurred in 2018.	➢ Participation of academics to advise epidemiological surveillance studies in the region of the disaster in 2019.
	➢ Renova Foundation challenged study results in 2020.	
Culture and Leisure	➢ Class-action lawsuit demanded that Samarco remove waste from areas used for leisure and affected cultural assets.	➢ One day after the disaster, a recommendation was issued for Vale to neutralize wastes to preserve historic and cultural heritage.
	➢ Consent decree not respected by Samarco.	
Community Participation	➢ Communities affected not included in discussions regarding reparations. Complaints that Renova prevented access to information.	➢ Participation of affected communities from the beginning. Hiring consultants to facilitate participation and explain content of agreements, lawsuits, and technical/legal jargon.
Tourism	➢ No news reports on tourism.	➢ Tourism always mentioned in combination with leisure and culture.

## Data Availability

Publicly available data sets were analyzed in this study. These data can be found here: https://sigdesastre.kinghost.net/; https://www.tjmg.jus.br/portal-tjmg/; https://www.mpmg.mp.br/; http://www.mpf.mp.br/ (accessed on 30 July 2021).
